# Neural correlates of risk perception: HIV vs. leukemia

**DOI:** 10.3389/fnbeh.2013.00166

**Published:** 2013-11-19

**Authors:** Alexander Barth, Ralf Schmälzle, Britta Renner, Harald T. Schupp

**Affiliations:** Department of Psychology, University of KonstanzKonstanz, Germany

**Keywords:** risk perception, HIV, leukemia, intuition, ERP

## Abstract

Field studies on HIV risk perception suggest that people may rely on impressions they have about the safety of their partner. Previous studies show that individuals perceived as “risky” regarding HIV elicit a differential brain response in both earlier (~200–350 ms) and later (~350–700 ms) time windows compared to those perceived as safe. This raises the question whether this event-related brain potential (ERP) response is specific to contagious life-threatening diseases or a general mechanism triggered by life-threatening but non-contagious diseases. In the present study, we recorded dense sensor EEG while participants (*N* = 36) evaluated photographs of unacquainted individuals for either HIV or leukemia risk. The ERP results replicated previous findings revealing earlier and later differential brain responses towards individuals perceived as high risk for HIV. However, there were no significant ERP differences for high vs. low leukemia risk. Rather than reflecting a generic response to disease, the present findings suggest that intuitive judgments of HIV risk are at least in part specific to sexually transmitted diseases.

## Introduction

Risk perception is a catalyst for protective health behaviors (Armitage and Conner, 2000; Renner and Schwarzer, 2003), but its mechanisms remain insufficiently understood. By and large, risk perception has been conceptualized as beliefs about the probability and severity of certain health hazards (Weinstein, 2000; Renner and Schupp, 2011). Specifically, the “risk as analysis” view holds that risk perception is based on a deliberate gauging of the likelihood that one will be affected by a negative event (e.g., being infected with HIV) and the severity of that event (e.g., lethal). However, recent theories of risk suggest that peoples’ understanding of risk derives more from an “intuitive sensing” than “deliberate analysis” (Loewenstein et al., 2001; Slovic and Peters, 2006). According to this perspective, the intuitive perception of risk is a rapid, automatic, incidental and affectively charged process, as opposed to much slower, controlled, voluntary, and cognitive consideration of probability and severity (see also Lieberman, 2000; Hodgkinson et al., 2008).

Risk perception in the context of HIV provides a highly relevant real-world example supporting the notion that risk perception builds on intuitive processing: Studies using focus groups and retrospective interviews with HIV positive people reveal that people usually have spontaneous impressions about risk—i.e., they often reported that they were convinced that their partners were safe (Maticka-Tyndale, 1991; Gold et al., 1992; Gold, 1993; Keller, 1993). These impressions about a potential partner’s HIV risk may make people prone to rely on “illusory control strategies” (Thompson et al., 2002), such as skipping condom use or selecting safe-looking partners. Recently, a new line of research examined the mechanisms behind these pervasive impressions of HIV risk by measuring neural responses while participants performed evaluations of HIV risk. Specifically, recording event-related brain potentials (ERPs) allows studying key features of intuitive processing, such as the fast and frugal processes that unfold during the first few hundred milliseconds after stimulus onset (e.g., the first sighting of a risky- or safe-looking person). Recent ERP studies of HIV risk perception revealed that differentiation between risky and safe individuals occurs early in the processing stream (< 300 ms) (Schmälzle et al., 2011, 2012; Renner et al., 2012). This early onset precedes systematic reasoning about health risks and supports the notion of intuitive as opposed to analytic processing, the latter being more laborious, time consuming and deliberate (Slovic and Peters, 2006). Moreover, one specific ERP component, the late positive potential (LPP), has been consistently observed as a cortical marker of affective significance across a wide range of stimuli (i.e., natural emotional scenes, facial expressions, and symbolic gestures) (Schupp et al., 2003; Kissler et al., 2006; Flaisch et al., 2009). Consistent with these findings, portraits of individuals perceived as highly risky elicited significantly larger LPPs compared to pictures of individuals believed to be safe (Schmälzle et al., 2011, 2012; Renner et al., 2012). Finally, there is also evidence suggesting that differences between risky and safe individuals reflect implicit processes, i.e., that they occur in the absence of a specific processing goal (Schmälzle et al., 2012). Overall, these results point to the intuitive nature of HIV risk perception.

The finding that HIV risk perception builds on intuitive impressions raises the question, what kind of information provides the foundation for these impressions? Due to the absence of overt signs, HIV cannot be reliably detected by visual inspection. Thus, we hypothesize that people base their judgments of risk on a high-risk stereotype containing a set of interrelated personality characteristics related to responsibility and trustworthiness (Renner and Schwarzer, 2003; Renner et al., 2012). Research on social person perception shows that inferences about such traits are remarkably efficient and could be obtained with minimal processing time. For instance, as little as 33 ms is sufficient to infer trust or threat (e.g., Bar et al., 2006; Willis and Todorov, 2006). Accordingly, when people report that they “just know” the risk posed by a certain individual, their risk ratings could actually reflect an implicit assessment of person characteristics related to the HIV risk stereotype. This perspective contains the critical assumption that intuitive impressions of HIV risk are domain-specific to implicit meaning structures of HIV, or life-threatening contagious diseases more generally (Bishop, 1991). An alternative possibility is that HIV risk perceptions reflect general and rather unspecific evaluations of health status.

The present study was designed to determine whether the quick and affect-related impressions of a person’s riskiness are specific to HIV or reflect a more generic process. The key characteristics of the HIV risk stereotype and intuitive sensing of HIV risk relate to the contagiousness dimension of disease representation (Bishop, 1991). As a contrast, leukemia was chosen as a sufficiently similar non-contagious disease with specific key differences (Skelton, 2006). First, leukemia is life-threatening, which rules out confounds related to differences in seriousness (i.e., contrasting a serious with a non-serious disease). Second, like HIV, the early stages of leukemia are not associated with overt symptoms. Third, both HIV and leukemia are diseases which are widely known but have a low incidence rate. Following this logic, we adapted the so-called AIDS-Leukemia paradigm, which originally was devised to examine attitudes towards individuals with HIV (Skelton, 2006). Participants in separate conditions were asked to evaluate the riskiness of unknown individuals for either HIV or leukemia (balanced across participants). Based on participants’ idiosyncratic judgments, safe and risky categories for HIV and leukemia were formed. The hypothesis that intuitive impressions of HIV risk are domain-specific to implicit meaning structures of sexually transmitted diseases predicts a significant interaction of Risk Level (low vs. high) and Disease (HIV vs. leukemia). With regard to HIV, the replication of previous findings of increased LPPs amplitudes for risky individuals and early differentiation between the risk categories (< 300 ms) were predicted. However, no significant effects were expected for low and high leukemia risk. Alternatively, if previous findings reflect a generic disease related process, both HIV and leukemia should elicit similar ERP differences for high and low risk individuals, resulting in a significant main effect of Risk Level.

## Materials and Methods

### Participants

Forty-two volunteers (aged 20–27 years , *M* = 22.5, SD = 2.0, 23 females) were recruited at the University of Konstanz. Participants received either 15 € or course credits as compensation. Six participants were excluded due to excessive EEG artifacts or insufficient trials. Participants provided written consent, which was approved by the Ethics Review Board of the University of Konstanz.

### Stimulus materials

The stimulus sets consisted of photographs of people in everyday scenes (Schmälzle et al., 2011; Renner et al., 2012). The photographs were retrieved with permission from a popular online photo-sharing community^1^ and each showed a single young adult of Caucasian appearance. Attire, socioeconomic cues, and context were intentionally included to provide naturalistic viewing conditions and facilitate impression formation. The two stimulus sets consisted of either 120 females or 120 males. Each set was complemented with 15 additional pictures comprising the task set for the implicit condition. Each participant evaluated the opposite sex to increase ecological validity.

### Procedure

A within-subject design was used to collect explicit ratings of HIV and leukemia risk in two separate rating task conditions, counterbalanced across participants. Both conditions were identical with regard to stimulus materials, stimulus presentation, and format of data collection. In each condition, the 120 (opposite sex) pictures were presented for 2 s in a random order for each participant and condition following a fixation cross (1 s). After a delay period of 1 s, participants were asked to evaluate how likely the presented person is HIV positive or has leukemia on a 7-point rating scale ranging from “very unlikely” [1] to “very likely” [7]. The next trial was initiated after an Inter-trial interval (ITI) of 3.5 s. To minimize possible order effects due to the novelty of the stimulus materials, participants viewed the stimulus materials before conducting the risk rating conditions.

### Categories of HIV and Leukemia risk

Stimuli were categorized separately for each condition and according to idiosyncratic risk ratings, whereby risk ratings of 1 to 3 were coded as “low risk” and 5 to 7 as “high risk”. There were no frequency differences between low (35.1 % and 35.2 %) and high risk (26.9 % and 27.5 %) categories for HIV and leukemia conditions. 2 (Disease) × 2 (Risk Level) ANOVA analysis revealed only a significant main effect of Risk Level, *F*(1,35) = 14.1, *p* < .001, while neither the main effect of Disease, *F*(1,35) = 0.2, *p* = .66 nor the interaction of Disease × Risk, *F*(1,35) = 0.1, *p* = .8, was significant. Initial analysis revealed no differences between male and female participants in HIV and leukemia risk judgments.

### Electrophysiological recording and data reduction

Electrophysiological data were collected using a 257-lead HydroCel Geodesic Sensor Net (EGI: Electrical Geodesics, Inc., Eugene, OR). The EEG was recorded continuously with a sampling rate of 250 Hz, with the vertex sensor as reference electrode, and on-line filtered from 0.1–100 Hz using Netstation acquisition software and EGI amplifiers. Impedances were kept below 50 kΩ, as recommended for this type of amplifier. Electromagnetic Encephalography Software (EMEGS; Junghöfer and Peyk, 2004) software was used for analysis. Data editing and artifact rejection were based on a method for the statistical control of artifacts specifically devised for analyzing dense sensor EEG recordings (Junghöfer et al., 2000). Preprocessing steps included low-pass filtering at 40 Hz, artifact detection, ocular artifact correction based on a multiple regression method (Miller et al., 1988), and bad sensor interpolation (Junghöfer et al., 2000). On average, 34.7 (SD = 7.5) out of 240 trials (14.5 %) were excluded with no difference between HIV and leukemia conditions (16.9 vs. 17.8, *t*(35) = 0.9, *p* = .35. Data reported were converted to an average reference and baseline-corrected for pre-stimulus (100 ms) ERP activity, and conversion to an average reference (Junghöfer et al., 2000).

#### ERP analysis

Two ERP components sensitive to HIV risk were identified by visual inspection and single sensor waveform analysis (Schupp et al., 2003). Careful inspection of the data revealed no ERP effects for the leukemia condition. Accordingly, two ERP components were scored and submitted to statistical analysis.

In a time interval between 200–300 ms post stimulus, the fronto-central component (low vs. high HIV risk) was scored including EGI sensors #144, 155, 164, 173, 181, 182, 183, 184, 185, 193, 194, 195, 196, 197, 202, 203, 204, 205, 206, 211, 212, 213, 214, 221, 222, 223 (right) and #40, 41, 42, 43, 44, 48, 49, 50, 51, 52, 53, 55, 56, 57, 58, 59, 60, 62, 63, 64, 65, 66, 69, 70, 71, and 72 (left). The effect appeared reversed in polarity over occipito-temporal sites and was assessed by collapsing across the following sensors #148, 156, 157, 158, 159, 165, 166, 167, 168, 174, 175, 176, 187 (right) and #113, 114, 115, 120, 121, 122, 123, 133, 134, 135, 136, 145, and 146 (left). The centro-frontal LPP component was indexed as mean activity from 390–510 ms comprising right (#9, 16, 17, 22, 23, 24, 29, 30, 35, 36, 40, 41, 42, 43, 44, and 49) and left (#4, 5, 6, 7, 14, 185, 186, 197, 198, 206, 207, 213, 214, 215, 223, and 224) EGI sensors.

The early ERP components were submitted to a repeated-measures ANOVA including the independent variables “Disease” (HIV vs. leukemia), “Risk Level” (low vs. high), “Location” (fronto-central vs. occipito-temporal), and “Laterality” (left vs. right). The late ERP component was entered in ANOVA analysis including the independent variables of “Disease”, “HIV Level”, and “Laterality”. Initial analyses included also the factor Gender. However, there were no higher-order interactions of Disease × Risk Level × Gender in the ERP analysis, and this factor was not further considered. Where appropriate, degrees of freedom were adjusted using the Greenhouse–Geisser method to correct for violations of sphericity.

## Results

### Explicit risk perception: HIV and Leukemia risk ratings

**Figure 1:**
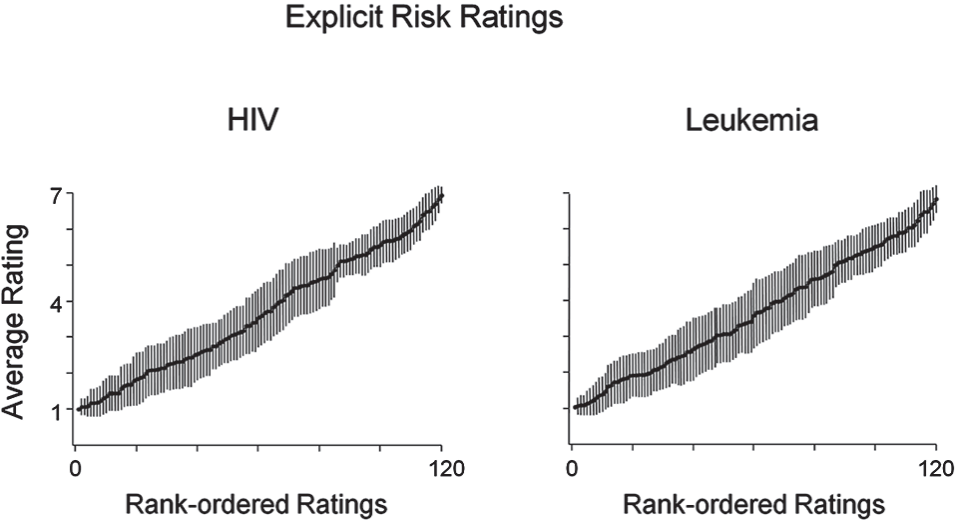
**Average ratings of HIV and leukemia risk.** Average ratings of HIV and leukemia risk and associated standard errors after rank-ordering each participant’s ratings by HIV and leukemia risk, respectively. Participants’ ratings of HIV and leukemia risk similarly varied across the full range of the scale (1—low risk; 7—high risk).

In a first step, risk rating distribution across both conditions was compared to confirm substantial variance. Taking an idiosyncratic perceptive, we first calculated the risk ratings’ variance and range of each participant’s risk ratings before ordering them by rank. In a second step, to illustrate the data at the group-level, we calculated mean responses for each rank across participants. As shown in Figure 1, mean HIV risk ratings increased from very low (minimum = 1) to very high (maximum = 6.94). Similarly, as shown in Figure 1, ratings of leukemia risk range from very low (minimum = 1.02) to very high risk (maximum = 6.83). These analyses demonstrate that the naturalistic stimuli produced broad variations both in perceived HIV and leukemia risk within and across participants. At the group level, HIV risk ratings (*M* = 3.67, SD = .39) and leukemia risk ratings (*M* = 3.68, SD = .45) were in the medium risk range and did not differ significantly, *t*(35) = −.084, *p* = .93. Furthermore, neither the minima or maxima, slope (*M* = .05 and *M* = .05) nor intercept (*M* = .79 and *M* = .69) of the rank ordered risk ratings for HIV and leukemia were significantly different, *t*(35) = −.61, *p* = .54 (slope) and *t*(35) = .81, *p* = .42 (intercept).

### Intuitive risk perception: ERPs

#### Fronto-central and occipito-temporal component (200–300 ms)

**Figure 2:**
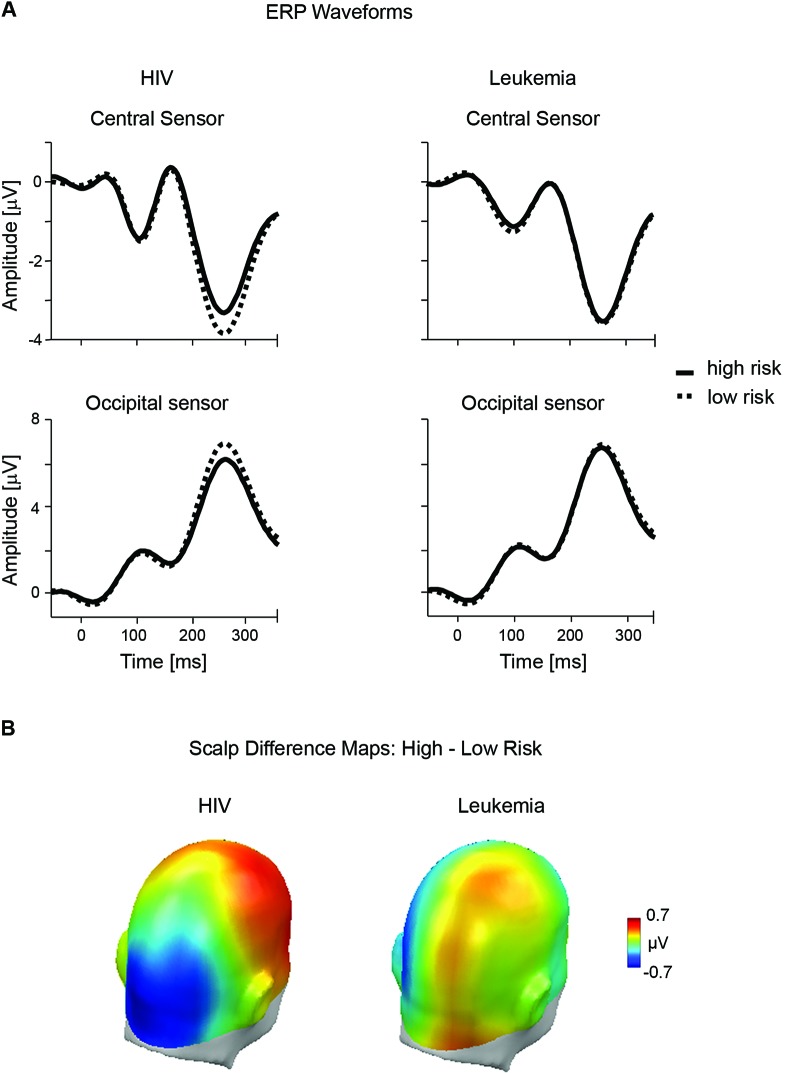
**Early ERP effects of risk for HIV and leukemia risk.** **(A)** ERP waveforms from representative central (#197) and occipital (#135) sensors contrasting high and low risk categories for HIV and leukemia. **(B)** The scalp potential map shows the topography of the difference between the high and low risk categories for HIV and leukemia risk averaged across the time window from 200–300 ms.

As illustrated in Figure 2A, the present study obtained evidence for a relatively early modulation of the ERP waveform by HIV risk. Overall, the ERP waveform presents a positive polarity over posterior sensors and a negative polarity over anterior sites. However, encoding risky stimuli resulted in a negative shift in the ERP waveform over occipito-temporal sensor regions and a corresponding positive shift over fronto-central sensor sites. The topography of the differential ERP activity (i.e., relative posterior negativity and anterior positivity) for high HIV risk is further illustrated by the calculation of difference maps (high —low HIV risk; see Figure 2B). Of most interest, as shown in Figure 2A, there was no difference in ERP waveforms between low and high risk categories for the leukemia condition. Substantiating these observations, the overall ANOVA analysis revealed a significant interaction for the factors Disease × Risk Level × Location, *F*(3,105) = 10.71, *p* < .01, partial *η*^2^ = 0.23. Accordingly, separate ANOVAs were calculated for both diseases.

For HIV, the ANOVA analysis revealed a significant interaction of Risk Level × Location, *F*(1,36) = 8.75; *p* < 0.01, partial *η*^2^ = 0.20, indicating that the effects of high and low HIV risk appeared with reversed polarity over fronto-central and occipito-temporal sites. A main effect of HIV risk, *t*(36) = 3.47, *p* < .01, partial *η*^2^ = 0.26, was observed over fronto-central leads, indicating a less negative potential for high HIV risk stimuli (*M* = − 2.02, SD = 1.37) compared to low HIV risk (*M* = −2.32, SD = 1.57). The HIV risk effect reversed in polarity over occipito-temporal sites, *t*(36) = 2.48, *p* < 0.05, partial *η*^2^ = 0.15. High HIV risk stimuli (*M* = 4.47, SD = 2.84) elicited a less positive potential compared to low HIV risk stimuli (*M* = 4.94, SD = 2.70). While the effect appeared to be more pronounced over right fronto-central sites, no effects involving the variable “Laterality” reached significance in these analyses.

For leukemia risk, ANOVA analysis revealed no significant interaction of Risk Level × Location, *F*(1,36) = 1.01; *p* = 0.32, partial *η*^2^ = 0.03. For exploratory reasons, separate analyses were conducted for fronto-central and occipito-temporal sensor clusters. There was no significant difference for high (*M* = 5.08, SD = 2.82) and low (*M* = 5.18, SD = 2.85) leukemia risk over occipito-temporal sites, *t*(36) = 0.46, *p* = 0.65, partial *η*^2^ < 0.01. However, a significant main effect of leukemia was observed over fronto-central leads, *t*(36) = 2.16, *p* < .05, partial *η*^2^ = 0.12. Interestingly, with a more negative potential for high leukemia risk stimuli (*M* = −2.25, SD = 1.33) compared to low leukemia risk stimuli (*M* = −2.10, SD = 1.49), the effect appeared with opposite polarity to the HIV risk modulation.

#### Centro-frontal component (390–510 ms)

**Figure 3:**
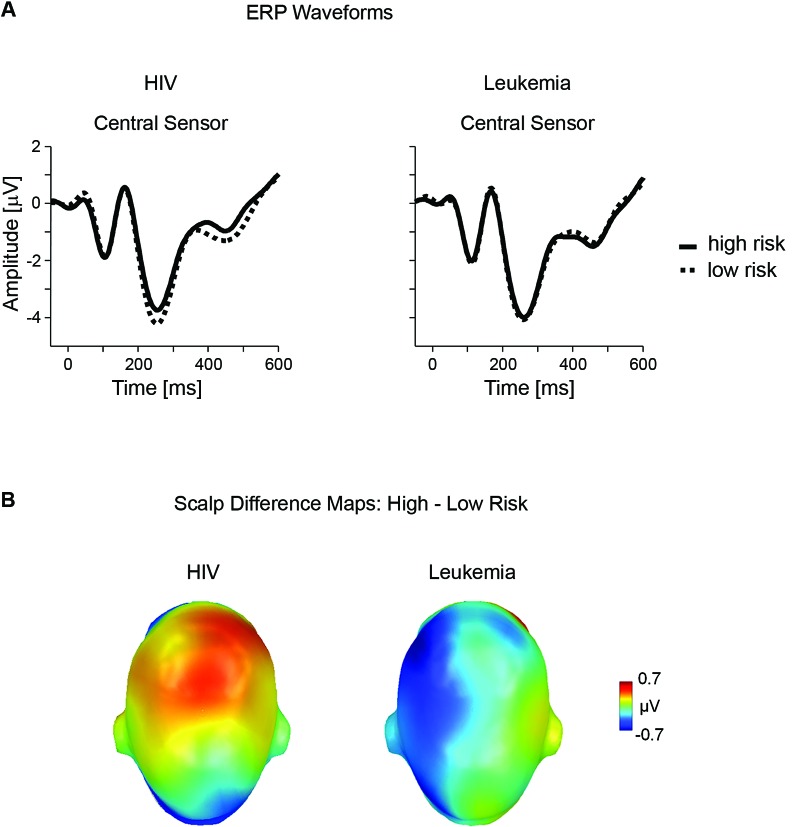
**Late ERP effects of risk for HIV and leukemia risk.** **(A)** ERP waveforms from representative central (#198) sensor contrasting high and low risk categories for HIV and leukemia. **(B)** The scalp potential map shows the topography of the difference between the high and low risk categories for HIV and leukemia risk averaged across the time window from 390–510 ms.

A second modulation of the ERP by perceived HIV risk status appeared in a time window between 390 and 510 ms over centro-frontal sensor sites. Differential ERP activity (high - low HIV risk), illustrated in Figure 3A, shows that the processing of risky stimuli is associated with a relative positive potential. Again, these effects were specific to the HIV condition and judgments of high leukemia risk were associated with relatively negative potential, unlike positive potential observed for high HIV risk. The significant interaction of Disease × Risk Level, *F*(1,35) = 6.17, *p* < .05, partial *η*^2^ < 0.15, was followed up by a separate analysis of both diseases.

For HIV, statistical analysis confirmed significant differences for high (*M* = −1.21, SD = 1.97) and low HIV risk stimuli (*M* = −1.53, SD = 1.89), *t*(36) = 2.00, *p* = .05, partial *η*^2^ = 0.10. For leukemia, high (*M* = −1.76, SD = 1.76) and low (*M* = −1.45, SD = 2.17) risk stimuli differed only at a marginal significance level, *t*(36) = 1.87, *p* = .07, partial *η*^2^ = 0.09, indicating however an opposite polarity as observed for HIV risk (see Figure 3B).

## Discussion

The present study contrasts the perception of HIV and leukemia risk. To shed light on their neural correlates, brain signals were recorded while participants evaluated pictures of unknown individuals for either HIV or leukemia risk. In line with previous findings, differential brain responses towards high vs. low HIV risk individuals were observed. In contrast, there were no significant ERP differences towards individuals with high vs. low leukemia risk.

The physical appearance of individuals provides a rich source of information and it has been widely acknowledged that inferences about personality characteristics based on first impressions are second nature to humans. However, it is far less widely recognized that such impressions might also extend to the domain of health risk perception and trigger feelings of risk or impulses towards disease avoidance. For instance, taking an evolutionary perspective, Schaller (2011) proposes a “behavioral immune system” that links perceivable signs of disease and infection to feelings of risk and avoidance behaviors, similar to the functions of the immune system. Avoiding an infectious disease provides an obvious advantage for survival and accordingly represents an adaptive mechanism. However, implicit processes linking person appearance cues to health risk are manifold. In some cases, these perceptual cues might be misleading since they are linked to perceived personality characteristics rather than actual signs of infection and disease. Specifically, research revealed that people may base the risk of a potential partner being infected by HIV on an intuitive mode of processing, i.e., the feeling of risk or safety, and ERP studies revealed corresponding neural correlates accompanying the differentiation between risky and safe HIV judgments. One interpretation of these findings is the assumption that implicit HIV risk stereotype knowledge contains personality characteristics amenable to snap judgments, which provide the basis for the intuitive sensing of risk or safety in the context of sexually transmitted diseases (Renner et al., 2012). This reasoning assumes that the brain correlates associated with HIV risk judgments are specific to sexually transmitted diseases rather than based on general tagging mechanisms of disease or illness. The current ERP results are in favor of such a disease-specific view of first impressions about HIV risk in that the differential processing of high and low risk was specific to HIV whereas no significant effects were found for leukemia.

With regard to the neural precursors of HIV risk judgments, the current results indicate that two ERP components, an early occipital negativity (200–300 ms) and a mid-latency central positivity (390–510 ms), were significantly larger for risky as compared to safe individuals. This is in line with previous findings (Schmälzle et al., 2011; Renner et al., 2012) and provides further support to the hypothesis of an intuitive mode of risk perception with regard to key features of intuition. Specifically, the risk as feeling hypothesis proposes a central role of affect in the intuitive sensing of danger and risk. In this respect, both ERP differentiations are similar to what is observed during ERP studies of emotional stimulus processing. Research with highly arousing emotional scenes such as erotica or mutilations as well as low and moderately arousing materials such as emotional faces, gestures, words, or clashing moral statements revealed these two ERP components to have similar topography, polarity, and latency (Junghöfer et al., 2001; Schupp et al., 2003, 2004; Kissler et al., 2007; Van Berkum et al., 2009; Flaisch et al., 2011). These findings suggest that risky-looking individuals elicit the brain signature of emotional significance, which is linked to selective visual attention. Furthermore, these findings support the notion of a negativity bias in that stimuli signaling danger are more effective in engaging affect processing than stimuli signaling safety (Cacioppo et al., 1999; Baumeister et al., 2001). A further characteristic of intuition is speed and fast processing time. The short time in which the brain discriminates between risky and safe individuals (~200 ms) clearly precedes the opportunity for systematic reasoning about health risks. Together, these findings support the fast and efficient processing of HIV risk related information.

While the ERP results support the hypothesis of the intuitive perception of HIV risk, a different pattern of results emerged for the perception of leukemia risk. Low and high risk for leukemia was not associated with a significant modulation of the ERPs. Specifically, there was no significant effect for the two ERP components differentiating high and low HIV risk categories or any other ERP component reliably differentiating between high and low leukemia risk. The lack of significant ERP modulation for leukemia risk seems not to be secondary to a lack of differentiation in reported risk as leukemia risk ratings showed similarly broad variations within and across participants. Interestingly, contrasting the risk judgments for both diseases revealed a remarkable similarity of data distribution with respect to mean values, variance, and range of risk ratings (Figure 1). However, the explicit risk ratings are in clear contrast to the ERP recordings, which indicate pronounced differences in the ERP waveforms accompanying risk judgments for HIV but not leukemia. To elicit the brain signature of affect processing, i.e., enhanced LPPs, and rapidly discriminate low and high HIV risk categories, the brain has to rely on stored memory representation, which supports fast and frugal heuristic processing during initial perceptual encoding. The finding that leukemia was not related to such ERP differences suggests that the memory representation leading to systematic ERP differences for high and low HIV risk is disease specific.

The present study is a first attempt to probe the structure and type of information supporting intuitive sensing of HIV risk. Lay disease representations (contagiousness and seriousness) (Bishop, 1991) were acknowledged by selecting a life threatening but not contagious disease. This variance in contagiousness captured a central characteristic of disease in order to discriminate whether the ERP correlates associated with high HIV risk are at least in part specific to HIV and sexually transmitted diseases or simply reflect a generic process. The life-threatening nature of leukemia also assured that the ERP differences are not secondary to differences in seriousness. Furthermore, leukemia is well known, minimizing the potential confound of the control condition being unknown to college students. Control questions, collected at the end of the study, revealed that all participants were familiar with the disease. Finally, HIV and leukemia are low-incidence diseases, minimizing the risk that pronounced differences in incidence may confound interpretation. In this respect, the findings reveal that the memory representations accessed by intuition are distinct for contagious and non-contagious diseases. Future studies may follow up these findings by contrasting different kinds of contagious diseases. The domain specific view proposed here would assume that the ERP differences related to HIV primarily occur for sexually transmitted diseases given that there is evidence for shared representations of a high risk stereotype among sexually transmitted diseases (Bishop, 1991; Renner and Schwarzer, 2003). Furthermore, the present study specifically selected leukemia for its lack of overt signs in the early stage. As such, it might be informative to contrast HIV with diseases associated with perceivable signs of infection to compare the neural correlates across different types of intuitive processes.

It has been suggested that a high-risk stereotype of HIV based on interrelated person characteristics provides the basis for intuitive judgments of HIV risk (Renner and Schwarzer, 2003; Renner et al., 2012). In particular, perceptions of HIV risk are strongly related to perceived lack of responsibility and low trustworthiness (Schmälzle et al., 2011; Renner et al., 2012). Several studies show that person characteristics, in particular trust, can be extracted quickly and with little effort, making people prone to form first impressions (Bar et al., 2006; Willis and Todorov, 2006). In a previous study, categorizing EEG data based on ratings of responsibility and trustworthiness showed similar ERP effects as obtained for HIV risk (Schmälzle et al., 2011), suggesting that HIV risk, trustworthiness, and responsibility share common meaning structures. Furthermore, a recent fMRI study of HIV risk perception (Häcker et al., 2010) revealed that high HIV risk was associated with increased activations in the insular cortex, a structure that has also been implicated in perceptions of trust (Castle et al., 2012). Notably, increased insular activations for high HIV risk were observed during implicit and explicit processing conditions and a similar finding was obtained in an earlier ERP study (Schmälzle et al., 2011). Likewise, implicit and explicit trustworthiness perceptions were related to the activation of the amygdala and insular cortex (Winston et al., 2002; Todorov and Engell, 2008). Thus, inferences about HIV risk and related person characteristics may build upon a partially shared representation associated with a general disposition for avoidance behavior (Todorov et al., 2008). Future work should explore which cues are utilized as signs of high or low HIV risk as well as a broader range of person characteristics such as trustworthiness, aggressiveness, sexual orientation, or attractiveness (e.g., Willis and Todorov, 2006; Rule and Ambady, 2008).

## Conclusion

HIV continues to be a serious challenge. To date, prevention remains the main strategy to dam rising infection rates. Therefore, it is indispensable to understand any barrier to effective preventive behaviors. Intuition about HIV risk may oppose consistent condom use by inducing a false sense of control. The present findings demonstrate that the heuristic processing underlying such intuitions is at least in part domain specific for sexually transmitted diseases.

## Conflict of interest statement

The authors declare that the research was conducted in the absence of any commercial or financial relationships that could be construed as a potential conflict of interest.

## Footnotes

^1^http://www.flickr.com
